# Cysteine-linked dimerization of BST-2 confers anoikis resistance to breast cancer cells by negating proapoptotic activities to promote tumor cell survival and growth

**DOI:** 10.1038/cddis.2017.68

**Published:** 2017-03-16

**Authors:** Wadie D Mahauad-Fernandez, Chioma M Okeoma

**Affiliations:** 1 Department of Microbiology, Carver College of Medicine, University of Iowa, Iowa City, IA, USA; 2 Interdisciplinary Graduate Program in Molecular and Cellular Biology (MCB), University of Iowa, Iowa City, IA, USA

## Abstract

Almost all breast tumors express the antiviral protein BST-2 with 67%, 25% and 8.2% containing high, medium or low levels of BST-2, respectively. Breast tumor cells and tissues that contain elevated levels of BST-2 are highly aggressive. Suppression of BST-2 expression reprograms tumorigenic properties of cancer cells and diminishes cancer cell aggressiveness. Using structure/function studies, we report that dimerization of BST-2 through cysteine residues located in the BST-2 extracellular domain (ECD), leads to anoikis resistance and cell survival through proteasome-mediated degradation of BIM—a key proapoptotic factor. Importantly, BST-2 dimerization promotes tumor growth in preclinical breast cancer models *in vitro* and *in vivo*. Furthermore, we demonstrate that restoration of the ECD cysteine residues is sufficient to rescue cell survival and tumor growth via a previously unreported pathway—BST-2/GRB2/ERK/BIM/Cas3. These findings suggest that disruption of BST-2 dimerization offers a potential therapeutic approach for breast cancer.

Although the attributes of BST-2 that orchestrate its tumorigenic behavior^1, 2, 3, 4^ are not well understood, the tumorigenic functions of BST-2 in mouse models of breast cancer are consistent with human clinical data.^3^ Various cellular mechanisms, including, cell to cell adhesion, anchorage-independent growth, migration and invasion have been associated with BST-2-mediated promotion of cancer.^1, 2, 3, 4^

Adhesion of cancer cells to components of the tumor microenvironment, such as extracellular matrix proteins, other cancer or stromal cells, is essential for cancer cells survival and growth.^5, 6, 7^ Cancer cell adhesion not only promotes cell clustering and tumor growth, it also facilitates inter- and intracellular signaling that results in cancer cell resistance to anoikis.^6, 7, 8^ The resultant net effect of cancer cell clustering is increased metastatic potential and decreased host survival.^9, 10^

Given that BST-2 in breast cancer cells mediates cancer cell adhesion and anchorage-independent growth,^3^ we hypothesized that BST-2 functions to promote tumor cell survival through inhibition of anoikis. Our data show that the cysteine residues in the extracellular domain (ECD) of BST-2 are required for cancer cells to resist anoikis and for tumors to grow.

## Results

### Comparative analysis of BST-2 protein in breast tumors

**Figure 1:**
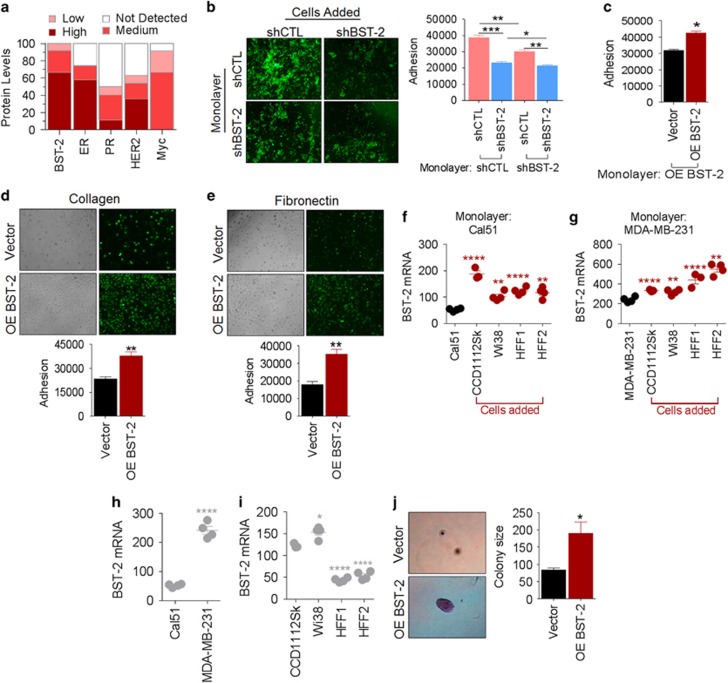
BST-2 regulates breast cancer cell adhesion and anchorage-independent growth. (**a**) *In silico* comparison of BST-2, estrogen receptor (ER), progesterone receptor (PR), human epidermal growth factor receptor 2 (HER2) and Myc protein levels in human breast tumor tissues. Levels were determined by immunohistochemistry (IHC) and data were downloaded from ProteinAtlas.org. (**b**) Images and quantification of adherence of PKH67Green-labeled 4T1 BST-2-expressing shControl (shCTL) and BST-2-suppressed (shBST-2) cells onto 4T1 shCTL and shBST-2 monolayers. (**c**) Quantification of adherence of PKH67Green-labeled MCF-7 cells expressing empty vector (Vector) or overexpressing wild-type BST-2 (OE-BST-2) onto MCF-7 cells overexpressing WT BST-2 (OE-BST-2). (**d** and **e**) Images and quantitation of adherence of PKH67Green-labeled MCF-7 cells expressing empty vector or OE-BST-2 on collagen-coated or fibronectin-coated plates. Adhesion was analyzed by florescent imaging and absorbance reading at 485/535 nm. The RFI is the adhesion rate presented as Adhesion. (**f** and **g**) Meta-analysis of BST-2 mRNA levels in Cal51 and MDA-MB-231 breast cancer cell lines co-cultured with or without CCD1112SK, Wi38, HFF1 or HFF2 fibroblast cell lines. (**h** and **i**) Meta-analysis of BST-2 mRNA levels in Cal51 and MDA-MB-231 breast cancer cell lines and CCD1112SK, Wi38, HFF1 and HFF2 fibroblast cell lines. Data used in panels (**f**) to (**i**) are from GEO dataset GSE41678.^38^ (**j**) Representative images and colony size of crystal violet-stained MCF-7 cell growth in soft agar assay showing anchorage-independent growth of Vector- or OE-BST-2-expressing MCF-7 cells. Colony diameters from five different fields of six different wells were measured following a 30-day transformation assay. The colony sizes were averaged and a percent calculated relative to MCF-7 Vector-expressing cells, which was set up to 100%. All experiments were repeated at least three times and similar results were observed. Error bars correspond to S.E.M. Significance was taken at *P<0.05, **P<0.01, ***P<0.001, ****P<0.0001

In agreement with previous meta-analysis of BST-2 mRNA,^3, 11^ assessment of BST-2 protein levels using Protein Atlas (http://www.proteinatlas.org) confirms presence of BST-2 protein in breast tumors (Figure 1a). The data show that more breast tumors contain elevated BST-2 protein compared with the levels of estrogen receptor, progesterone receptor, HER2 or Myc. These data suggest that BST-2 may have a significant role in breast cancer and could be a valuable therapeutic target.

### BST-2 mediates adhesion of breast cancer cells to components of the tumor microenvironment

To investigate the role of BST-2 on cellular interactions, we performed an adhesion assay using previously reported^3^ 4T1 cells stably expressing scrambled shRNA (shCTL—BST-2 expressing) or BST-2-targeting shRNA (shBST-2—BST-2 suppressed) (Supplementary Figure 1). Results show that BST-2-expressing incoming cancer cells efficiently adhere to monolayers of shCTL cells compared with BST-2-suppressed cells. When the monolayer consists of BST-2-suppressed cells, adhesion of BST-2-suppressed cells was also reduced (Figure 1b, image and bar). Additional experiments using MCF-7 cells overexpressing (OE) empty vector or wild-type BST-2 show that BST-2 is required for cell to cell or cell to ECM interactions. Although MCF-7-vector cells adhere marginally to the monolayer, OE-BST-2 enhances MCF-7 adherence (Figure 1c). In addition, MCF-7-OE-BST-2 cells efficiently adhere to collagen (Figure 1d) and fibronectin (Figure 1e) pre-coated plates compared with MCF-7-vector cells. These data indicate that resident cancer cells anchored on supportive structure or incoming cancer cells in suspension require BST-2 for efficient adhesion.

### Adhesive interaction between fibroblasts and breast cancer cells upregulate BST-2 expression

To further evaluate the significance of BST-2-mediated adhesive interactions, we show that adhesion between monolayers of Cal51 (Figure 1f) or MDA-MB-231 (Figure 1g) and several strains of fibroblasts significantly upregulates BST-2 expression in the cancer cells. The magnitude of BST-2 upregulation may depend on the basal level of cancer cell BST-2 because BST-2 level (Figure 1h) and the magnitude of fibroblast-mediated BST-2 upregulation (Figures 1f and g) are lower in Cal51 compared with MDA-MB-231. In addition, fibroblasts contain varying levels of BST-2 (Figure 1i), but their BST-2-inducing capability is comparable (Figures 1f and g). These data indicate that interactions between cancer cells and fibroblasts may regulate cancer cell BST-2 and may promote cellular reprograming.

### BST-2 expression is required for efficient growth of breast cancer cells in suspension

To assess the biological relevance of BST-2-mediated adhesive interactions, we first examined the effects of BST-2 on anchorage independency and spheroid formation. MCF-7-OE-BST-2 cells produced larger spheroids compared with MCF-7-vector cells (Figure 1j). These data indicate that BST-2 promotes survival and growth of cancer cells in suspension; suggesting that breast tumor cells that are anchorage independent due to high levels of BST-2 may undergo anoikis in circulation in the absence of BST-2.

### BST-2 dimers are present in breast cancer cells and dimerization is regulated by ECD cysteine residues

**Figure 2:**
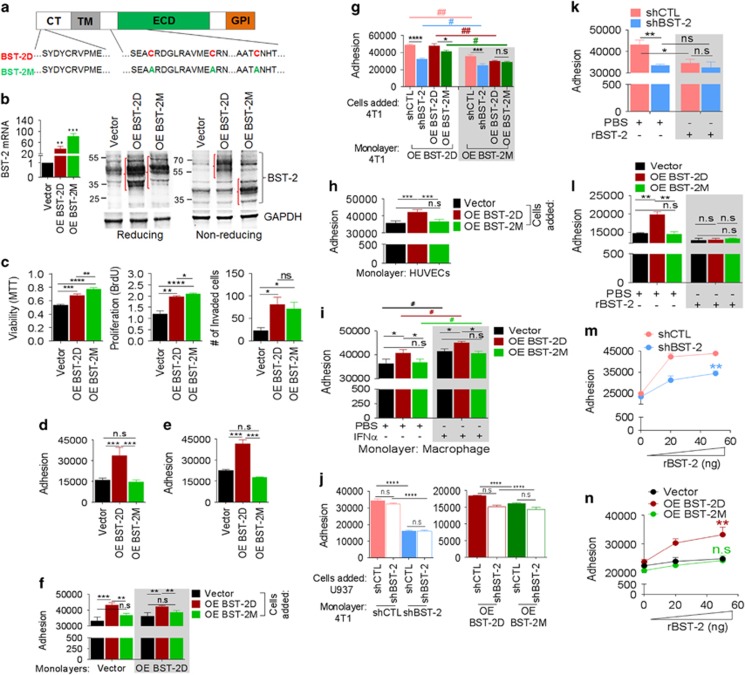
Covalent dimerization of BST-2 mediates breast cancer cell adhesion. (**a**) BST-2 protein containing the cytoplasmic tail (CT), transmembrane domain (TM), ECD and the glycophosphatidylinositol anchor (GPI). Underneath are the different amino-acid residues color-coded to match the variant of BST-2 following substitution of cysteine residues (red Cs; designated OE-BST-2D for dimers) with alanine residues (green As; designated OE-BST-2M for monomers). (**b**) RT-qPCR and western blot (reducing and non-reducing) showing levels and variants of BST-2 in MCF-7 cells. Red brackets depict shift in BST-2 for BST-2 dimers—OE-BST-2D or monomers—OE-BST-2M. GAPDH was used as loading control and for normalization of RT-qPCR data presented as fold change. (**c**) Viability, proliferation and invasion analysis of MCF-7 cells expressing BST-2. Absorbances were read at 590 nm (MTT) and 450 nm (BrdU). Invaded cells stained with crystal violet were quantified from average of five different fields. (**d**–**n**) Adherence of: (**d**) Vector, OE-BST-2D, OE-BST-2M MCF-7 cells onto (**d**) collagen-coated plates and (**e**) fibronectin-coated plates. (**f**) Vector, OE-BST-2D, OE-BST-2M MCF-7 cells onto monolayers of Vector or OE-BST-2D MCF-7 cells. (**g**) shCTL, shBST-2, OE-BST-2D, OE-BST-2M 4T1 cells onto monolayers of OE-BST-2D (white background) and OE-BST-2M (gray background) 4T1 cells. (**h**) Vector, OE-BST-2D, OE-BST-2M MCF-7 cells onto HUVEC monolayers. (**i**) Vector, OE-BST-2D, OE-BST-2M MCF-7 cells onto PBS or IFN*α*-treated macrophage monolayers. (**j**) shCTL and shBST-2 U937 monocytes onto monolayers of shCTL, shBST-2, OE-BST-2D or OE-BST-2M 4T1 cells. (**k**) shCTL and shBST-2 4T1 cells onto rBST-2 or PBS-pretreated 4T1 shCTL monolayers. (**l**) Vector, OE-BST-2D, OE-BST-2M MCF-7 cells onto rBST-2 or PBS-pretreated MCF-7 OE-BST-2D monolayers. (**m**) shCTL and shBST-2 4T1 cells onto rBST-2-coated plates. (**n**) Vector, OE-BST-2D, OE-BST-2M MCF-7 cells onto rBST-2-coated plates. Before adhesion, incoming cells were labeled with PKH67Green. Adhesion was analyzed as RFI at 485/535 nm. Experiments were repeated at least three times with similar results. Error bars=S.D. for RT-qPCR and S.E.M. for other data. Significance= *P<0.05, **P<0.01, ***P<0.001, ****P<0.0001. Stars=outside and #=within-group significance. NS, not significant

Owing to the effective role of BST-2 in cell adhesion, we evaluated the structural property of BST-2 that has a role in cell adhesion. We engineered MCF-7 cells (Figure 2a) stably expressing WT BST-2 that is predominantly dimer (OE-BST-2D) and dimerization-deficient BST-2 that is expressed as monomers (OE-BST-2M). PCR analysis shows efficient expression of BST-2D and BST-2M mRNA (Figure 2b), whereas western blots confirm presence of BST-2D or BST-2M in these cells (Figure 2b). Functionally, BST-2D and BST-2M increase viability, proliferation and invasion of MCF-7, albeit with subtle differences (Figure 2c).

### BST-2 dimers mediate adhesion of breast cancer cells to components of the tumor microenvironment

Here, we assessed whether the variant of BST-2 in cancer cells is critical for BST-2:BST-2 interactions that mediate cell to cell and/or cell to matrix adhesion.^3^ As expected, OE-BST-2D but not OE-BST-2M significantly increases adhesion of cancer cells to collagen (Figure 2d) and fibronectin (Figure 2e), despite comparable expression (Figure 2b). OE-BST-2D cells have increased ability to bind low BST-2-expressing and high BST-2-expressing cells (Figure 2f). In contrast, OE-BST-2M cells have reduced ability to bind cells, irrespective of the level of BST-2 on the monolayer (Figure 2f). Further analyses show that adhesion of cancer cells expressing different levels and variants of BST-2 increases when shBST-2 monolayers are forced to express BST-2D but not BST-2M (Figure 2g).

The significance of BST-2 dimerization was further appreciated in experimental settings where adhesive interactions of cancer cells with other cells were examined. Compared with OE-BST-2D cells, OE-BST-2M cancer cells have reduced ability to bind endothelial cells—HUVECs (Figure 2h) and immune cells—macrophages (Figure 2i, white background) and monocytes (Figure 2j). Induction of BST-2 in macrophages with the BST-2 agonist, IFN*α*,^12^ results in increased adherence of cancer cells expressing the different variants of BST-2 to IFN*α*+ macrophages compared with IFN*α*- macrophages (Figure 2i, white and gray backgrounds). The increased adherence of OE-BST-2M cells to IFN*α*+ macrophages could be attributed to enhancement of endogenous BST-2D. Furthermore, monocytes, irrespective of their level of BST-2, adhere efficiently to monolayers of shCTL cells compared with shBST-2 cells (Figure 2j, pink and blue bars). The effect of BST-2 is dependent on the variant of BST-2, as monocytes expressing shCTL and shBST-2 adhere more efficiently to BST-2D-expressing monolayers (Figure 2j). These data indicate that the level and variant of BST-2 in cancer cells may determine the rate of immune cell adherence.

Next, we performed adhesion in the presence and absence of recombinant BST-2 (rBST-2). Results show that rBST-2 efficiently blocks adhesion of BST-2-expressing cancer cells but has no effect on adhesion of BST-2-suppressed cells (Figure 2k), indicating that BST-2 is responsible for the observed adhesion. Furthermore, rBST-2 specifically blocks adhesion of OE-BST-2D cells but has no effect on OE-BST-2M cells (Figure 2l), confirming that the variant of BST-2 (D or M) is crucial in cancer cell adhesion.

We confirmed the role of BST-2 dimerization on adhesion by seeding equivalent numbers of cells on rBST-2 pre-coated plates. Compared with shCTL cells, shBST-2 cells were unable to adhere efficiently to rBST-2-coated plates (Figure 2m). Importantly, OE-BST-2D increases cell adherence, whereas OE-BST-2M did not (Figure 2n). These data suggest that recombinant human BST-2 binds to both murine and human BST-2 in cancer cells and blocks cancer cell to cancer cell adhesion.

### BST-2 dimerization regulates anchorage independency

**Figure 3:**
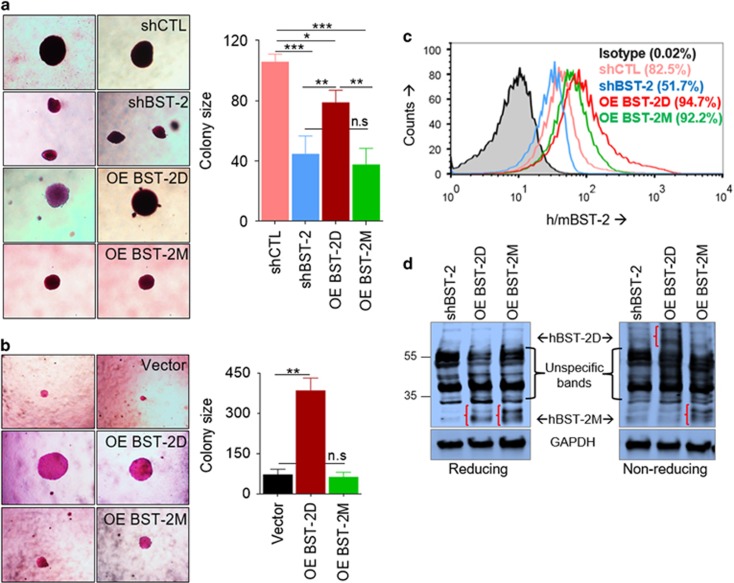
Covalent dimerization of BST-2 is important for anchorage-independent growth of breast cancer cells. (**a**) Representative images and quantitation of growth of 4T1 cells expressing different levels and variants of BST-2 on soft agar for 30 days. (**b**) Representative images and quantitation of growth of Vector, OE-BST-2D or OE-BST-2M MCF-7 cells on soft agar for 30 days. Clones were stained with crystal violet and imaged at 10X. To calculate colony size, the diameters of colonies from five different fields were measured, averaged and a percent calculated relative to either shCTL for 4T1 cells or Vector for MCF-7 cells, which were set to 100%. (**c**) FACS analysis of levels of variants of BST-2 on the surface of 4T1 shBST-2 cells. Numbers in parenthesis correspond to mean fluorescence intensity of BST-2 expression presented as percent. (**d**) Western blot analysis of total BST-2 protein under reducing and non-reducing conditions. Red brackets depict shift in BST-2D but not in BST-2M sizes under different conditions. Experiments were repeated at least three times with similar results. Error bars represent S.E.M. and significance was taken at *P<0.05, **P<0.001 and ***P<0.001. NS, not significant

As BST-2 dimerization is critical for cellular and matrix interactions, we showed that BST-2 dimerization is crucial for colony formation and anchorage-independent growth. As expected, 4T1 shCTL cells form significantly larger colonies compared with 4T1 shBST-2 cells (Figure 3a). OE-BST-2D but not OE-BST-2M efficiently rescues colony formation in shBST-2 cells (Figure 3a), indicating that BST-2 dimerization is required for growth of cancer cells independent of anchor. These findings were confirmed with MCF-7 cells (Figure 3b). The difference in the ability of OE-BST-2D and OE-BST-2M cells to grow in suspension is not because of the level of BST-2 (Figures 3c and d) but can be attributed to the variant of BST-2 (Figure 3d, red brackets—BST-2 shifts in non-reducing gels). These data indicate that BST-2 expressed as dimers may endow cancer cells the ability to cluster, survive and grow in suspension—a characteristic of aggressive epithelial-derived tumor cells.

### BST-2 dimerization promotes adherent-independent survival of cancer cells by inhibiting anoikis

**Figure 4:**
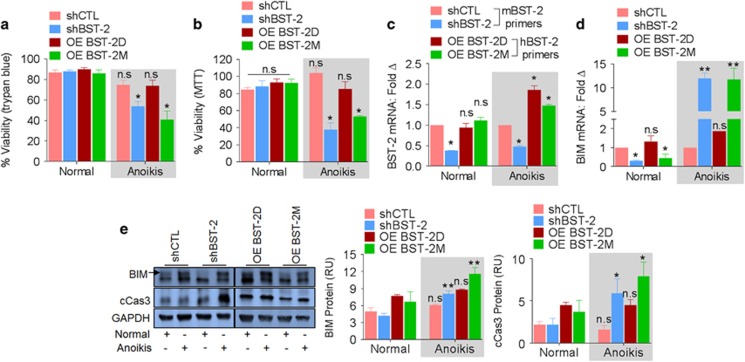
Dimerization of BST-2 protects cells from anoikis. (**a**) Trypan blue analysis of viability (survival) of 4T1 cells expressing different levels and variants of BST-2 and cultured under adherent (normal) or anoikis conditions at 37 °C for 48 h. (**b**) MTT analysis of viability (survival) of 4T1 cells expressing different levels and variants of BST-2 and cultured under adherent (normal) or anoikis conditions at 37 °C for 48 h. (**c**) RT-qPCR analysis of BST-2 mRNA in 4T1 cells expressing different levels and variants of BST-2 and cultured under adherent (normal) or anoikis conditions at 37 °C for 48 h. (**d**) RT-qPCR analysis of BIM mRNA in 4T1 cells expressing different levels and variants of BST-2 and cultured under adherent (normal) or anoikis conditions at 37 °C for 48 h. GAPDH was used as internal control and for normalization of RT-qPCR data. (**e**) Western blot analysis and quantitation (relative units (RUs)) of BIM and cleaved Cas3 protein levels in 4T1 cells expressing different levels and variants of BST-2 and cultured under adherent (normal) or anoikis conditions at 37 °C for 48 h. GAPDH was used as loading control. Experiments were repeated at least three times with similar results. Error bars correspond to S.E.M. for viability assays and protein quantification and to S.D. for RT-qPCR data. Significance was taken at *P<0.05 and **P<0.01. NS, not significant

If BST-2 dimerization is involved in protection of cancer cells from anoikis, cells expressing BST-2D will survive under anoikis conditions. Indeed, following poly-HEMA-mediated induction of anoikis, shBST-2 cells have significant reduction in viability compared with shCTL cells (Figures 4a and b). OE-BST-2D but not OE-BST-2M rescues viability of shBST-2 cells (Figures 4a and b). The inability of BST-2M cells to survive under anoikis conditions is due to the variant of BST-2 because BST-2 mRNA is higher in BST-2M cells compared with shBST-2 cells (Figure 4c).

Next, we assessed the levels of molecules implicated in anoikis, including BIM and caspase-3 (Cas3). BIM mRNA was suppressed in shBST-2 and OE-BST-2M cells compared with shCTL and OE-BST-2D cells in normal conditions (Figure 4d). However, upon induction of anoikis, the level of BIM mRNA (Figure 4d), BIM protein and cleaved Cas3 (Figure 4e) increased. These observations were confirmed using MCF-7 cells where OE-BST-2M cells in suspension showed reduced survival, increased BIM mRNA, and increased BIM and cCas3 proteins (Supplementary Figures 2a-c), despite elevated BST-2 mRNA (Supplementary Figure 2d). These results suggest that BST-2 dimerization promotes cancer cell survival by inhibiting anoikis.

### BST-2 dimerization results in phosphorylation of BST-2 in cancer cells

**Figure 5:**
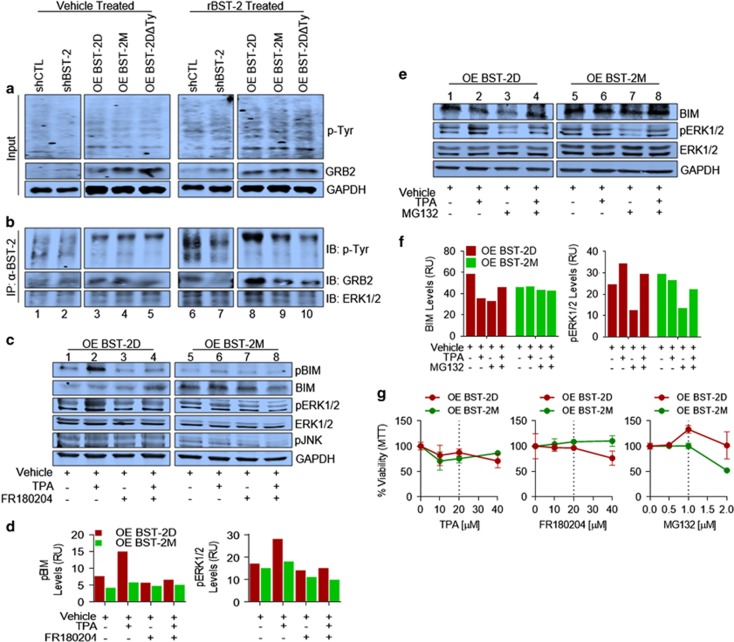
BST-2 dimerization increases ERK1/2 activation, BIM phosphorylation and proteasome-mediated degradation. (**a**) Western blot analysis of total protein lysates from 4T1 cells expressing different levels (shCTL and shBST-2) and variants (OE-BST-2D, OE-BST-2M or OE-BST-2DΔTy) of BST-2 and pretreated with vehicle or rBST-2. Blots were probed with antibodies against phosphorylated tyrosines (p-Tyr), GRB2 and GAPDH. (**b**) Immunoprecipitation and western blot analysis of shCTL, shBST-2, OE-BST-2D, OE-BST-2M and OE-BST-2DΔTy 4T1 cells pretreated with vehicle or rBST-2. Immunoprecipitation was performed with anti-BST-2 antibodies and western blot performed with p-Tyr, GRB2 and ERK1/2 antibodies. (**c**) Total protein from 4T1 OE-BST-2D and OE-BST-2M cells treated with DMSO (Vehicle), 20 nM of TPA (survival signal), 20 *μ*M of FR180204 (ERK1/2 inhibitor) or TPA + FR180204 (TPA/FR180204) were used for western blot detection of phosphorylated BIM S69 (pBIM), total BIM (BIM), phosphorylated ERK1 and ERK2 (pERK1/2), total ERK1/2 (ERK1/2), phosphorylated JNK T183/Y185 (pJNK) and GAPDH. (**d**) Quantitation of pBIM and pERK1/2 from the gel shown in **c**. Data are presented as relative units (RUs) for protein level normalized to GAPDH. (**e**) Total protein from 4T1 OE-BST-2D and OE-BST-2M cells treated with DMSO (Vehicle), 20 nM of TPA (survival signal), 1 *μ*M of MG132 (proteasome inhibitor) or TPA + MG132 (TPA/MG132) were used for western blot detection of pBIM, pERK1/2, ERK1/2 and GAPDH. (**f**) Quantitation of BIM and pERK1/2 from the gel shown in **e**. Data are presented as RUs for protein level normalized to GAPDH. (**g**) Analysis of 4T1 OE-BST-2D and OE-BST-2M viability (MTT assay) following treatment with different concentrations of TPA, FR180204 and MG132 for 24 h. Dotted line shows the concentrations of the different small molecules used to treat cells. Experiments were repeated at least three times with similar results. Error bars correspond to S.E.M.

To explore the mechanism by which BST-2 promotes cellular interactions, we showed that dimerization of BST-2 molecules activates BST-2. Western blot analysis of input protein following exposure of cells to vehicle or rBST-2 shows that the levels of GAPDH, phosphor-tyrosine (p-Tyr), and growth factor receptor-bound protein 2 (GRB2) are similar (Figure 5a). In contrast, immunoprecipitation with anti-BST-2 antibody reveals that BST-2 is tyrosine phosphorylated in cancer cells in a manner that is dependent on BST-2 dimerization (Figure 5b). These data suggest that BST-2D-expressing cells contain activated and more function-relevant BST-2.

To determine the role of activated BST-2 in cancer cells, we started by investigating the level of GRB2 – a docking protein that binds to phospho-tyrosine residues of activated receptors and recruits ERK1/2 to the signaling complex. The level of GRB2 remained the same in quiescent cells (Figure 5b). But upon BST-2 activation, higher GRB2 and ERK1/2 were bound to phospho-BST-2 in shCTL and OE-BST-2D cells (Figure 5b). Remarkably, GRB2 and ERK1/2 bound to BST-2 in OE-BST-2M cells did not increase upon BST-2 activation (Figure 5b).

We confirmed that BST-2 is tyrosine phosphorylated using a mutant form of BST-2 that is able to form dimers but the cytoplasmic tail tyrosine residues at positions 6 and 8 had been substituted with alanine residues (OE-BST-2DΔTy). The level of anti-BST-2-precipitated p-Tyr, GRB2 and ERK1/2 did not change between vehicle and rBST-2-treated OE-BST-2DΔTy cells (Figure 5b), suggesting that phospho-Y6/Y8 recruits GRB2.

### BST-2 dimerization results in ERK-mediated BIM phosphorylation

As BIM protein is degraded by the proteasome following its phosphorylation by kinases – ERK1/2 and JNK,^13, 14, 15, 16^ we used 12-O-tetradecanoylphorbol-13-acetate (TPA) to induce survival signal and phosphorylate BIM. TPA treatment phosphorylates ERK1/2 and BIM in OE-BST-2D cells but not in OE-BST-2M cells, although total ERK1/2, BIM and phosphorylated JNK were unchanged from vehicle-treated cells (Figure 5c). These data indicate that activation of ERK1/2 and phosphorylation of BIM are dependent on BST-2 dimerization.

To confirm that ERK1/2-mediated BIM phosphorylation is BST-2 dimerization dependent, we exposed OE-BST-2D and OE-BST-2M cells to FR180204 – ERK1/2 specific inhibitor.^17^ Compared with vehicle, FR180204 had no effect on protein levels in OE-BST-2D and OE-BST-2M cells (Figures 5c and d). However, inhibition of ERK1/2 activity in OE-BST-2D but not OE-BST-2M cells treated with TPA results in increased total BIM and reduced pERK1/2 and pBIM (Figures 5c and d). Importantly, total ERK1/2 and pJNK were not affected by FR180204 (Figure 5c).

### BST-2 dimerization induces proteasomal degradation of BIM

As BST-2D downregulates BIM, we examined whether this downregulation occurs via proteasomal degradation. Although TPA activates/phosphorylates ERK1/2 and decreases BIM levels in OE-BST-2D cells, MG132 treatment results in accumulation of BIM in TPA-treated cells (Figures 5e and f). Importantly, TPA, MG132 or TPA/MG132 has no effect on the levels of pERK1/2 and BIM in OE-BST-2M cells (Figures 5e and f). The concentration of inhibitors used were non-toxic (Figure 5g) and not responsible for the observed BST-2-independent reduction of pERK1/2 in MG132-treated cells (Figures 5e and f). Together, results in Figure 5 suggest that BST-2 dimerization promotes ERK1/2 activation, BIM phosphorylation/degradation and inhibition of Cas3 activation that culminate in enhanced anoikis resistance – a phenotype required by cancer cells to survive in circulation.

### CTC clusters of metastatic breast cancer patients are enriched in BST-2

**Figure 6:**
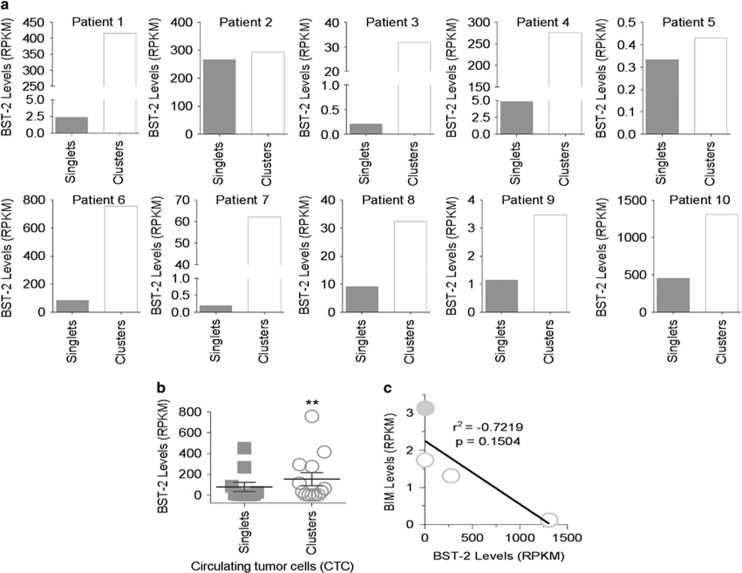
BST-2 mRNA in CTCs inversely correlates to BIM mRNA. (**a**) Meta-analysis of the levels of BST-2 mRNA (presented as RPKM units) present in CTC singlets (singlets) and CTC clusters (clusters) isolated from the blood of 10 different patients with metastatic breast cancer. (**b**) Average BST-2 mRNA levels from CTC singlets and clusters of 10 patients. (**c**) Correlative analysis of BST-2 and BIM levels in CTC singlets (filled circle) and clusters (open circle). The *r*^2^ value depict strong inverse correlation between BST-2 and BIM in CTCs. Lack of significance is attributed to lack of enough data points. Samples with RPKM values of zero were excluded from the study. Data were from GEO dataset GSE51827.^22^ Error bars correspond to S.E.M. Significance was taken at **P<0.01

The clinical significance of BST-2-mediated cell clustering and survival was evaluated using data from a publicly available dataset^9^ to compare the levels of BST-2 in circulating tumor cells (CTCs). Intrapatient comparison of BST-2 in CTC singlets *versus* CTC clusters shows that 8 of 10 patients have CTC clusters that express higher BST-2 than their respective CTC singlets (Figure 6a). On the average, CTC clusters express higher (~2 fold) BST-2 compared to CTC singlets (Figure 6b). Further analysis shows that BIM RNA inversely correlates with BST-2 RNA in CTCs (Figure 6c), supporting the findings in Figures 4d and e and further suggest that BST-2 may facilitate cancer cell clustering, thus protecting cancer cells from hemodynamic shear stress in circulation.

### BST-2 dimerization regulates the growth of triple-negative breast cancer cells in mice

**Figure 7:**
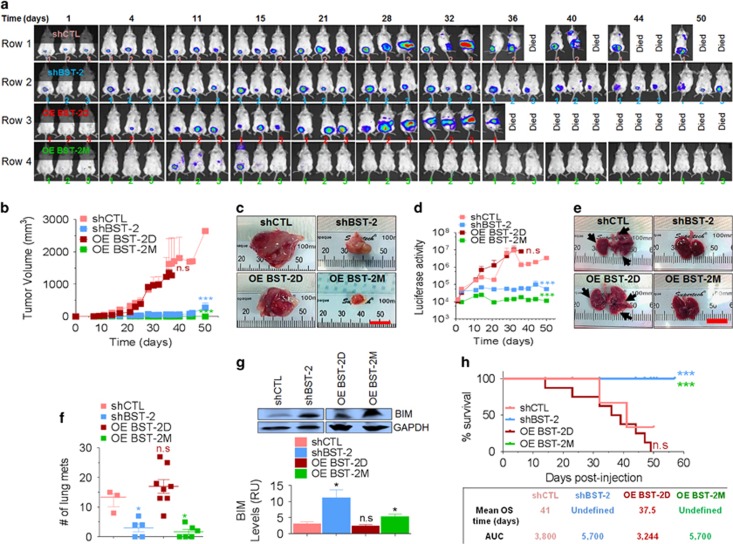
BST-2 dimerization is required for breast tumor growth in a preclinical model of triple-negative breast cancer. (**a**) Representative images of tumor cells tracked *in vivo* at different time points with IVIS imaging following injection of mice with luciferase expressing shCTL (*n*=3), shBST-2 (*n*=8), OE-BST-2D (*n*=8) or OE-BST-2M (*n*=8) 4T1 cells. The numbers denote day following tumor cell injection. (**b**) TV over time computed as TV=0.5 (length × width^2^) from mice bearing shCTL, shBST-2, OE-BST-2D or OE-BST-2M 4T1 tumors. (**c**) Gross images of primary mammary tumors obtained at necropsy. (**d**) Luciferase activity tracked with IVIS imaging from mice bearing shCTL, shBST-2, OE-BST-2D or OE-BST-2M 4T1 tumors. (**e**) Representative gross images of lungs. Lung nodules (macrometastases, arrow heads) are visible in shCTL and OE-BST-2D tumor-bearing mice. (**f**) Quantitative representation of lung nodules from mice bearing shCTL, shBST-2, OE-BST-2D or OE-BST-2M 4T1 tumors. (**g**) Western blot detection and quantitation (relative units (RUs)) of BIM and GAPDH proteins in total lung protein lysates from mice bearing shCTL, shBST-2, OE-BST-2D or OE-BST-2M 4T1 tumors. GAPDH was used as internal control and for normalization of BIM levels. (**h**) Kaplan–Meier survival plot of mice bearing shCTL, shBST-2, OE-BST-2D or OE-BST-2M 4T1 tumors. 4T1 shCTL, OE-BST-2D and shBST-2 tumors grew to varying sizes. Only three of eight OE-BST-2M tumors grew. Scale bar=10 mm. Error bars correspond to SEM. Significance was taken at *P<0.05 and ***P<0.001. AUC, area under the curve; NS, not significant; OS, overall survival

Compared with shCTL, shBST-2 cells have a significant decrease in primary tumor growth (Figures 7a and c). Analysis of tumor volume (TV) and final tumor mass show that OE-BST-2D but not OE-BST-2M rescues tumor growth potential of shBST-2 cells (Figures 7a and c). OE-BST-2D and shCTL but not shBST-2 and OE-BST-2M cells efficiently metastasize as evidenced by increased luciferase expression over time (Figures 7a and d). In addition, we observed increase spontaneous pulmonary metastases of shCTL and OE-BST-2D but not shBST-2 and OE-BST-2M tumors (Figures 7e and f). The decrease in lung metastasis in shBST-2 and OE-BST-2M tumor-bearing mice could be attributed to smaller primary tumors, although the effect of BST-2 on primary tumor is distinct from its effect on lung metastasis.^3^ Alternatively, dimerization-competent OE-BST-2D cancer cells could associate with BST-2-expressing lung-associated cells, and such association may protect cancer cells from apoptosis. Indeed, BIM levels in the lungs of shCTL and OE-BST-2D tumor-bearing mice were significantly reduced compared with the level in shBST-2 and OE-BST-2M tumor-bearing mice (Figure 7g). These data indicate that BST-2 dimerization is required for tumor growth and that disruption of BST-2 dimerization may render metastatic cells susceptible to apoptotic insult in the lungs.

According to Kaplan–Meier's survival analysis, growth of shCTL and OE-BST-2D tumors culminates in death with mean overall survival of 37.5 and 41.0 days for OE-BST-2D and shCTL tumor-bearing mice, respectively (Figure 7h). At variance, the mean overall survival for shBST-2 and OE-BST-2M tumor-bearing mice was undefined (Figure 7h). Together, these data suggest that disruption of BST-2 dimerization may serve to prevent tumor growth and metastatic colonization of the lungs, thus increasing overall survival of tumor-bearing hosts.

**Figure 8:**
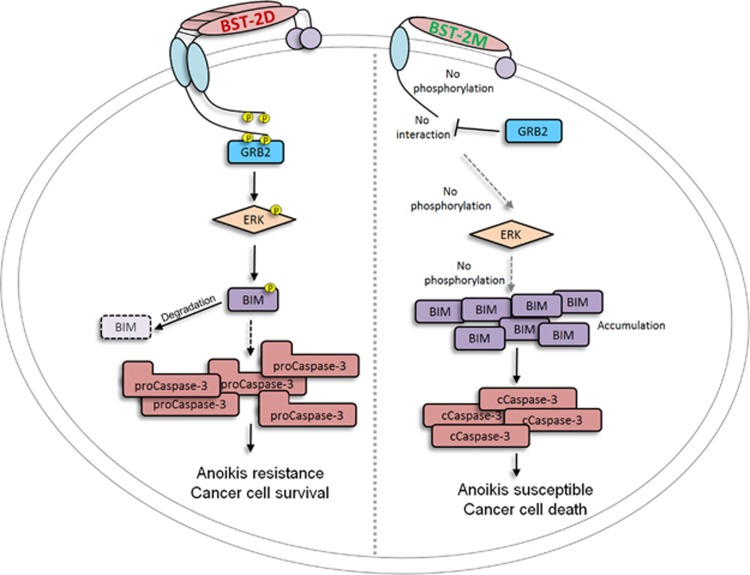
Hypothetical model of BST-2-mediated anoikis resistance. Wild-type dimer-forming BST-2 (BST-2D) in cancer cells is activated upon cell to cell or cell to ECM interaction. BST-2 activation results in phosphorylation of the cytoplasmic tail (CT), presumably at the tyrosines residues located at positions 6 and 8. Other phosphorylation events independent of these tyrosines are possible. Phosphorylated BST-2 recruits GRB2 (an adaptor protein that recognize p-Tyr), facilitating activation of yet to be identified kinase(s), such as Src or Ras, which in turn phosphorylates ERK. Phospho-ERK then phosphorylates BIM resulting in subsequent proteasomal degradation and removal of BIM. In the absence of BIM, mitochondrial membranes remain intact and pro-Cas3 is not cleaved and activated (cCaspase-3). The end result is that cancer cells overcome anoikis, survive under harsh conditions and grow/metastasize

On the basis of these observations, we propose a new model for cancer cell survival and growth in which dimeric BST-2 orchestrates pro-adhesive and anti-anoikis stimuli (Figure 8). The principle of this new model is that dimeric BST-2 allows interaction between cancer cells and the tumor microenvironment that promotes the survival, growth and metastasis of tumor cells.

## Discussion

Here we provide evidence for structural and molecular link between BST-2 and breast cancer by highlighting the following:

First, the BST-2 ECD cysteine residues mediate formation of BST-2 dimers in cancer cells. Previous studies demonstrated the effect of BST-2 dimers in protection against viral infection.^18^ However, these studies did not evaluate the involvement of BST-2 dimerization in altering cancer cell behavior. Our study identifies BST-2 dimerization as critical in the promotion of cancer cell adhesion. We found that cancer cells expressing dimeric BST-2 efficiently adhere to other cancer cells, potential stromal cells, and ECM proteins. Thus, cancer cells expressing BST-2 dimers may serve as a target or docking sites for other cells and ECM proteins to bind to tumors. The interaction between BST-2-expressing primary tumor cells and other resident stromal cells may regulate the expression of other factors in secondary organs, thus conditioning metastatic sites for subsequent arrival of tumor cells, especially tumor cells that express dimeric BST-2. It remains to be determined how BST-2 mediates the adhesion of cancer cells to components of the ECM. Perhaps, the cysteine residues involved in BST-2 dimerization may associate with cysteine residues found on fibronectin type II domain.^19^

Second, similar to the role of BST-2 in adhesion, the ability of BST-2 to promote survival and growth of cancer cells in suspension is controlled by BST-2 dimerization. Cancer cell adhesion is intricately related to the ability of such cells to survive in suspension. We report that cells expressing BST-2 dimers and not monomers activate intracellular signaling that result in the degradation of BIM and blockade of Cas3 activation, culminating in anoikis resistance and cell survival. Therefore, BST-2:BST-2 dimerization may transmit survival signals or suppress proapoptotic factors in breast cancer cells, creating a microenvironment that allows cells to grow independent of anchor. In our studies, we identified BST-2/GRB2/ERK/BIM/Cas3 as an important pathway in anoikis evasion by breast cancer cells. This BST-2-directed cell reprograming allows cancer cells to survive in circulation. Evidently, BST-2 is present in circulating breast cancer cells, and levels are elevated in cells that circulate as clusters. Whether BST-2 is directly linked to cancer cell clustering and survival in human blood is yet to be determined. Meta-analysis of data from CTCs shows an inverse correlative association between BST-2 and BIM. This concept was experimentally validated with breast cancer cells *in vitro* and with lung tissues from tumor-bearing mice. Of note, the tumorigenic activity of BST-2 dimerization is operative across species (mouse and human) and is independent of the aggressive nature of the cells.

Third, our study extends our knowledge of the molecular mechanism of anoikis evasion and the positive impact of the proteasome on tumor growth. The loss of cell viability and growth arrest observed *in vitro* and *in vivo* following expression of monomeric BST-2 is dependent on blockade of GRB2 recruitment and ERK1/2 activation, proteasomal degradation of BIM and activation of Cas3. Whether or not other anti- or pro-apoptotic factors are involved is yet to be determined. Also unknown are the kinases that catalyze and co-ordinate this complex BST-2/GRB2/ERK/BIM/Cas3 pathway. Activation of ERK1/2 in cells expressing BST-2 dimers may be orchestrated by serine/threonine kinases, such as Src or Ras known to phosphorylate ERK.^20, 21^ In addition to accumulation of BIM protein, BIM mRNA was upregulated in cells expressing reduced levels of BST-2 or monomers of BST-2. It is unclear how BST-2 dimerization can lead to reduced BIM at the RNA level. In immune cells, BST-2 is negatively regulated by MYD88/PI3K.^22^ Possibly in cancer cells, BST-2 may activate PI3K to phosphorylate FOXO3A—a transcription factor that induces BIM expression^23, 24^ upon its dephosphorylation and nuclear translocation.^25^ Although the identity of the BST-2 tyrosine residues that are phosphorylated is yet to be revealed, it is known that the cytoplasmic tail of BST-2 contains two tyrosine residues at positions 6 and 8 that become phosphorylated upon virus-mediated BST-2 activation.^26^ Possibly, these or other tyrosines present in the different domains of BST-2 are phosphorylated. Indeed, *in silico* analysis using the PPSP software (http://ppsp.biocuckoo.org/)^27^ revealed that BST-2 contains four phosphorylatable tyrosines at positions 6, 8, 153 and 154.^28^ Aside from tyrosines, the cytoplasmic tail of BST-2 contains phosphorylatable serines and a threonine.

Fourth, we provide evidence that cells expressing monomeric BST-2 are unable to grow in the mammary gland. As monomeric BST-2 is deficient in adhesion and anchorage independency, it is possible that these cells were unable to make contact with each other or with mammary gland resident cells. The lack of increased metastatic tumor growth in the lungs persuades us to speculate that tumor cells expressing monomeric BST-2 may alter the tumor environment landscape by changing the type of immune cells that are recruited to the tumor microenvironment because of changes in the expression of signaling cytokines and chemokines.^29, 30, 31^ It is also possible that cells expressing monomeric BST-2 may not survive in circulation thus limiting the number of cancer cells that may reach metastatic sites. Noteworthy, although expression of BST-2M in cells almost completely repressed tumor formation, shBST-2 cells (containing low levels of BST-2D) were able to form primary tumors, albeit small. Although the reason for BST-2M-mediated repression of tumor growth is yet to be determined, it is possible that functionally distinct signals may be elicited by BST-2M and shBST-2 cells and that BST-2M signals may have a negative growth effect on tumor cells (autocrine). It is also plausible that BST-2M signals may be transmitted to other distal cells to inhibit cell growth (paracrine).

In summary, we have demonstrated how BST-2 activity shapes the function of breast cancer cells. We identify BST-2/GRB2/ERK/BIM/Cas3 as the pathway regulating BST-2-mediated cancer cell adhesion, anoikis resistance, anchorage-independency, cell survival and growth. Our findings may motivate development of new targeted treatments based on disruption of BST-2 homodimerization in tumors.

## Materials and Methods

### Cell lines

The murine triple-negative breast cancer cell line-4T1 and the luminal A breast cancer cell line—MCF-7, respectively, are kind gifts from Drs. Lyse Norian and Weizhou Zhang of the University of Iowa, Iowa City, IA, USA. All cells were maintained according to ATCC guidelines (Manassas, VA, USA).

### Animals

Five-week-old female BALB/cAnNCr mice purchased from Harlan (Indianapolis, IN, USA) were used. Tumor-bearing mice were killed when they became moribund. TV was calculated as: TV=0.5(length × width^2^).^32^ Experiments involving mice were approved by the University of Iowa Animal Care and Use Committee (IACUC).

### Mice injections and live animal imaging

Orthotopic mammary tumors were generated by implanting 300 000 cancer cells into the 10th mammary fat pad of 5-week-old female mice. Before imaging, mice were anesthetized, weighed and injected intraperitoneally with d-luciferin (Sigma-Aldrich, St. Louis, MO, USA). Mice were imaged using the Xenogen IVIS three-dimensional optical imaging system (Caliper Life Sciences, Hopkinton, MA, USA). Luciferase expression was quantified with Living Image Software (Caliper Life Sciences). Primary tumors were weighted and photographed post-mortem. Pulmonary nodules were quantified by manual counting.

### Generation of BST-2-overexpressing cancer cells

MCF-7 cells, which contain low levels of endogenous BST-2^3^ or 4T1 shBST-2 cells in which endogenous mouse BST-2 was downregulated,^3^ were stably transfected with either empty pcDNA3.1 (Vector for MCF-7 cells or shBST-2 for 4T1 cells), pcDNA3.1 containing dimerization-competent wild-type human BST-2 (BST-2D) or pcDNA3.1 containing dimerization mutant BST-2 in which cysteine residues at positions 53, 63 and 91 were replaced with alanine residues (BST-2M). These BST-2 constructs are a kind gift from Dr. John Guatelli of UCSD (La Jolla, Ca, USA) and Dr. Klaus Strebel of NIH (Bethesda, MD, USA).^18, 33^ Lipofectamine 2000 (Life Technologies, Carlsbad, CA, USA) was used for the transfections and the amounts used were adjusted according to the manufacturers' instructions. Transfected cells were selected with G418 at 500 *μ*g/ml and stable cells were used in all experiments. Note that shBST-2 is a shRNA specific for mouse BST-2 and does not affect the expression of human BST-2.

### Assessment of BST-2 protein expression and phosphorylation

Western blots were performed as previously described.^34, 35, 36^ Briefly, protein extracts from MCF-7 or 4T1 cells expressing variants of BST-2 (Vector, OE-BST-2D, OE-BST-2M, shCTL, shBST-2, OE-BST-2D and OE-BST-2M) were isolated and assayed under reducing (*β*-ME + heat) and non-reducing (heat only) conditions as previously described.^18^ For BST-2 dimerization and activation/phosphorylation studies, equivalent numbers (300 000 cells) of shCTL, shBST-2, OE-BST-2D, OE-BST-2M and OE-BST-2DΔTy cells were seeded in six-well plates. Four hours later, cells were treated with 200 ng per well of rBST-2 or equivalent volume of vehicle for 1 h. Equivalent concentrations of total proteins from the cells were used to immunoprecipitate BST-2 using anti-BST-2 antibodies (AIDS reagents program, Germantown, MD, USA). Immunoprecipitates were separated and probed with anti-phospho-tyrosine, anti-GRB2, and anti-ERK1/2 antibodies (Cell Signaling Beverly, MA, USA). The species-appropriate IRDye secondary antibody was used followed by detection with the Odyssey Infrared Imaging System (LI-COR Biosciences, Lincoln, NE, USA).

### Evaluation of BST-2 surface protein

Approximately, 1 × 10^6^ 4T1 cells were stained with either APC-conjugated anti-human BST-2 antibody (BioLegend, San Diego, CA, USA) or appropriate immunoglobulin Gs (IgGs) for 1 h at 4 °C. Cells were washed and stained with 7-AAD viability dye (BioLegend) for 15 min. Using FACS Calibur flow cytometer (BD, San Jose, CA, USA), at least 10 000 events were collected per sample. FACS data were analyzed by Flowjo software (TreeStar, Ashland, OR, USA).

### Assessment of RNA levels

Isolation of RNA was accomplished using a RNeasy mini kit (Qiagen, Venlo, Netherlands) according to the manufacturer's instructions. For cDNA synthesis, equivalent amounts of RNA treated with DNase I (Qiagen) were reverse-transcribed with high capacity cDNA reverse transcription kit (ABI, Carlsbad, CA, USA). The cDNA was amplified with target-specific primers. Quantitative reverse transcription real-time qPCR (RT-qPCR) was carried out using ABI 7500 FAST thermal cycler. Primers used: GAPDH-forward: 5′-CCCCTTCATTGACCTCAACTACA-3′, reverse: 5′-CGCTCCTGGAGGATGGTGAT-3′ mouse BST-2-forward: 5′-TCAGGAGTCCCTGGAGAAGA-3′, reverse: 5′-ATGGAGCTGCCAGAGTTCAC-3′ human BST-2 RT^2^ qPCR primer assays (SA-Biosciences, Frederick, MD, USA) as well as hBST-2-forward: 5′-AGAAGGGCTTTCAGGATGTG-3′, reverse: 5′-CTTTTGTCCTTGGGCCTTCT-3′ BIM-forward: 5′-ATCGGAGACGAGTTCAACGA-3′, reverse: 5′-TGCCTTCTCCATACCAGACG-3′ and Cas3-forward: 5′-CAAAACCTCAGTGGATTCAAAA-3′, reverse: 5′-CCCATTTCAGGATAATCCATTT-3′.

### Cell to cell adhesion assay

Cells of interest were grown to form a confluent monolayer in 96-well plates. In all, 20 000 cancer cells of interest were labeled with PKH67Green fluorescent cell linker (Sigma-Aldrich). Labeled cells were added to the appropriate monolayers and allowed to incubate for 4 h. Non-adhered cells were washed off with PBS and plates were read at 485 nm/535 nm (excitation/emission) wavelengths using a Tecan Infinite M200 Pro plate reader (Tecan, Maennedorf, Switzerland) to determine the rate of adhesion. Values are represented as relative fluorescence intensity (RFI) or as percentage.

### Cell to ECM protein adhesion assay

Wells of a 96-well plate were coated with 50*μ*L of 50 *μ*g/ml collagen or fibronectin (Sigma-Aldrich) and incubated at 37 °C for 2 h. Nonspecific sites were blocked with 40 *μ*l of 2 mg/ml bovine serum albumin in PBS and then wells were washed once with PBS. In all, 20 000 PKH67Green (Sigma-Aldrich) labeled cancer cells (MCF-7 Vector, BST-2D or BST-2M or 4T1 shCTL or shBST-2 cells) were added to pre-coated wells and allowed to adhere for 4 h. Non-adhered cells were washed off with PBS and plates were read using a Tecan plate reader as described in the previous paragraph.

### Blockade of cell to cell adhesion

BST-2D-overexpressing MCF-7 cells or 4T1 shCTL cells were plated to confluency in a 96-well plate and blocked with water (Vehicle) or 200 ng/well of rBST-2 (Sino Biological Inc., Beijing, China) for 4 h. Cell monolayers were washed twice with PBS. 25 000 PKH67Green-labeled MCF-7 cells expressing Vector, OE-BST-2D or OE-BST-2M were added to MCF-7 cell monolayers. On the other hand, 25 000 PKH67Green 4T1 cells expressing shCTL or shBST-2 were added to 4T1 shCTL monolayers. Cells were allowed to adhere for 4 h at 37 °C. Plate was washed twice with PBS and fluorescence was read with a Tecan plate reader as described previously.

### Assessment of cancer cell viability and determination of small molecule IC_50_

A total of 10 000 MCF-7 cells expressing Vector, OE-BST-2D or OE-BST-2M or 4T1 cells expressing shCTL, shBST-2, OE-BST-2D, OE-BST-2M or OE-BST-2DΔTy were seeded in 96-well plates for 24 h. Cells were then left untreated or treated with 0, 0.5, 1 or 2 *μ*M of MG132; 0, 10, 20, 40 *μ*M of FR180204; or 0, 10, 20, 40 *μ*M of TPA. These cells were then incubated with 20 *μ*l/well of 5 mg/ml MTT reagent for 3.5 h followed by removal of media, addition of 150 *μ*l per well of MTT solvent (0.1% NP-40 and 4 mM HCl in isopropanol) and rocking for 15 min to determine the effect of different BST-2 constructs in cancer cell viability and to determine the IC_50_ of the different small molecules used. Absorbance at 590 nm was read using a Tecan Infinite M200 Pro plate reader.

### Induction of cell survival and analysis of the signal transduction pathway

Equivalent numbers (300 000 cells) of relevant cells were seeded on six-well plates and treated with DMSO (Vehicle), 20 nM of the survival signal TPA (Sigma-Aldrich), 1 *μ*M of the proteasome inhibitor MG132 (Sigma-Aldrich), a combination of TPA and MG132 (TPA/MG132), 20 *μ*M of the ERK1/2 kinase inhibitor FR180204 (Sigma-Aldrich), or a combination of TPA and FR180204 (TPA/FR180204) for 24 h following IC_50_ determination. Equivalent concentrations of total proteins from the cells were separated on a PAGE-gel and probed with an anti-BST-2 antibody (AIDS reagents program), anti-cleaved Cas3, anti-BIM, and anti-GAPDH antibodies (Santa Cruz Biotechnology, Dallas, TX, USA), as well as with anti-ERK1/2, anti-pERK1/2, anti-pJNK, anti-pAKT S473 and anti-pBIM antibodies (Cell Signaling). The species-appropriate IRDye secondary antibodies were used followed by detection with the Odyssey Infrared Imaging System (LI-COR Biosciences).

### Evaluation of cancer cell proliferation

A total of 10 000 relevant cells were seeded in 96-well plates for 24 h. Bromodeoxyuridine or 5-bromo-2′-deoxyuridine (BrdU) (Calbiochem, Billerica, MA, USA) assay was carried out according to the manufacturer's instructions. Absorbance at 450 nm was read using a Tecan Infinite plate reader.

### Colony formation assay

In all, 24-well plates were coated with 500 *μ*l of 0.5% agar and allowed to solidify.^3^ Following, 4T1 shCTL, shBST-2, OE-BST-2D or OE-BST-2M and MCF-7 Vector-, OE-BST-2D- or OE-BST-2M-expressing cells were plated at 1250 cells per well in 500 *μ*l of 0.35% agarose. In all, 250 *μ*l of complete RPMI was added on top of the agar layer. Growth medium was replaced twice a week and cells were allowed to form colonies for 30 days. Colonies were stained with crystal violet and imaged using a Nikon Eclipse Ti microscope adjusted with a Nikon digital sight camera (Nikon, Tokyo, Japan). The diameters of colonies from five different fields were measured, averaged and a percent calculated relative to either shCTL for 4T1 cells or Vector for MCF-7 cells, which was set to 100%.

### Invasion assay

The apical chamber of 24-well cell culture inserts (Merck Millipore, Billerica, MA, USA) were coated with 1.5 mg/ml of Matrigel (100 *μ*l) (Sigma-Aldrich) and allowed to solidify for 3 h. A total of 250 000 MCF-7 cells expressing Vector, OE-BST-2D or OE-BST-2M were starved for 6 h, suspended in serum-free medium and were plated on top of the Matrigel layer. In all, 600 *μ*l of culture medium containing 30% FBS and 5 *μ*g/ml fibronectin (Sigma-Aldrich) was added to the basal chamber of the unit and cells were allowed to invade through the membranous barrier for 24 h at 37 °C. Noninvasive cells were washed off; invasive cells were fixed with 4% PFA, permeabilized with 100% methanol, labeled with Giemsa stain and imaged. Images were processed using ImageJ software (NIH, Bethesda, MD, USA). Cells from five different fields were blind counted and averaged.

### Experimental induction and analysis of anoikis

U-bottom 96-well plates were coated with 50 *μ*l of sterile 95% ethanol or 50 *μ*l of 12 mg/ml poly-HEMA in 95% ethanol (Sigma-Aldrich) and allowed to dry for 72 h under the hood as previously described.^37^ Poly-HEMA prevents cells from attaching to the plastic. Following, 4T1 shCTL, shBST-2, OE-BST-2D or OE-BST-2M; and, MCF-7 Vector-, OE-BST-2D- or OE-BST-2M-expressing cells were seeded at 20 000 cells per well. Plates were centrifuged at 1200 × *g* for 10 min and then incubated at 37 °C for 48 h. Cells were collected to test cell viability using Trypan blue (Life Technologies) and a MTT assay (Life Technologies). The rest of the cells were pelleted and kept at −20 °C until used for RNA and protein isolation.

### Meta-analysis

The publically available Gene Expression Omnibus (GEO) data set GSE51827,^9^ which contains RNAseq data from CTCs singlets and clusters isolated from metastatic breast cancer patients was used to determine the levels of BST-2 mRNA. RPKM units were calculated using the formula: RPKM=(10^9^ × read counts)/(total mapped reads × exon length). Intrapatient comparisons were performed by plotting CTC singlets BST-2 levels along with CTC clusters BST-2 levels of the same patient. Correlation analyses of BIM and BST-2 levels were performed with all samples excluding patient samples whose BIM or BST-2 levels were zero. Moreover, for analyses of BST-2 expression in human mammary cancer epithelial cells (Cal51 and MDA-MB-231) co-cultured with different strains of human fibroblasts (CCD1112SK, Wi38, HFF1 and HFF2), the GEO dataset GSE41678^38^ were used. All data points were included in the analyses.

### Statistics

Statistical analysis of significant differences was performed with unpaired *t-*test assuming Gaussian distribution with Welch's correction or using a non-parametric Kolmogorov–Smirnov test (GraphPad Prism software, San Diego, CA, USA). Error bars represent S.D. for transcript data and S.E.M. for other data. Kaplan–Meier survival plots were analyzed using the Gehan–Breslow–Wilcoxon test (GraphPad Prism software). A probability (*P*) value of 0.05 or lower was considered significant.
