# Supplementary material for: Cysteine-linked dimerization of BST-2 confers anoikis resistance to breast cancer cells by negating proapoptotic activities to promote tumor cell survival and growth
Source: Cell Death Dis. 2017 Mar 16;8(3):e2687–. doi: 10.1038/cddis.2017.68 (PMC5386562; doi:10.1038/cddis.2017.68)
Supplement: Supplementary Figures [file cddis201768x1.doc]

Figure legend

**Supplemental Figure 1. BST-2 downregulation in 4T1 cells using an shRNA.** (a) BST-2 RNA levels in 4T1 cells stably expressing a scramble shRNA control (shControl, shCTL) or a BST-2-targeting shRNA (shBST-2). GAPDH was used as loading control and for normalization of RT-qPCR data presented as fold change. (b) FACS analysis of levels of BST-2 on the surface of 4T1 cells expressing shControl or shBST-2. Numbers in parenthesis correspond to mean fluorescence intensity of BST-2 expression presented as percent. Error bars correspond to standard deviation. Significance was taken at P<0.01**.

**Supplemental Figure 2.** **BST-2 dimerization renders MCF-7 cells resistant to anoikis via downregulation of BIM**. (a) Trypan blue analysis (left) and MTT analysis (right) of viability (survival) of MCF-7 cells expressing variants of BST-2 and cultured under adherent (Normal) or anoikis conditions at 37°C for 48 hours. (b) RT-qPCR analysis of BIM mRNA in MCF-7 cells expressing variants of BST-2 and cultured under adherent (Normal) or anoikis conditions at 37°C for 48 hours. (c) Western blot analysis and quantitation (relative units=RU) of BIM and cleaved Caspase-3 (cCas3) protein levels in MCF-7 cells expressing different levels and variants of BST-2 and cultured under adherent (Normal) or anoikis conditions at 37°C for 48 hours. (d) RT-qPCR analysis of BST-2 mRNA in MCF-7 cells expressing variants of BST-2 and cultured under adherent (Normal) or anoikis conditions at 37°C for 48 hours. GAPDH was used as internal control and for normalization of RT-qPCR and Western blot data. Experiments were repeated at least three times with similar results. Error bars correspond to SEM for viability assays and protein quantification and to SD for RT-qPCR data. Significance was taken at P<0.05* and P<0.01**. n.s = not significant. OE = Overexpression.
