# Supplementary figures and images for: Cysteine-linked dimerization of BST-2 confers anoikis resistance to breast cancer cells by negating proapoptotic activities to promote tumor cell survival and growth
Source: Cell Death Dis. 2017 Mar 16;8(3):e2687–. doi: 10.1038/cddis.2017.68 (PMC5386562; doi:10.1038/cddis.2017.68)

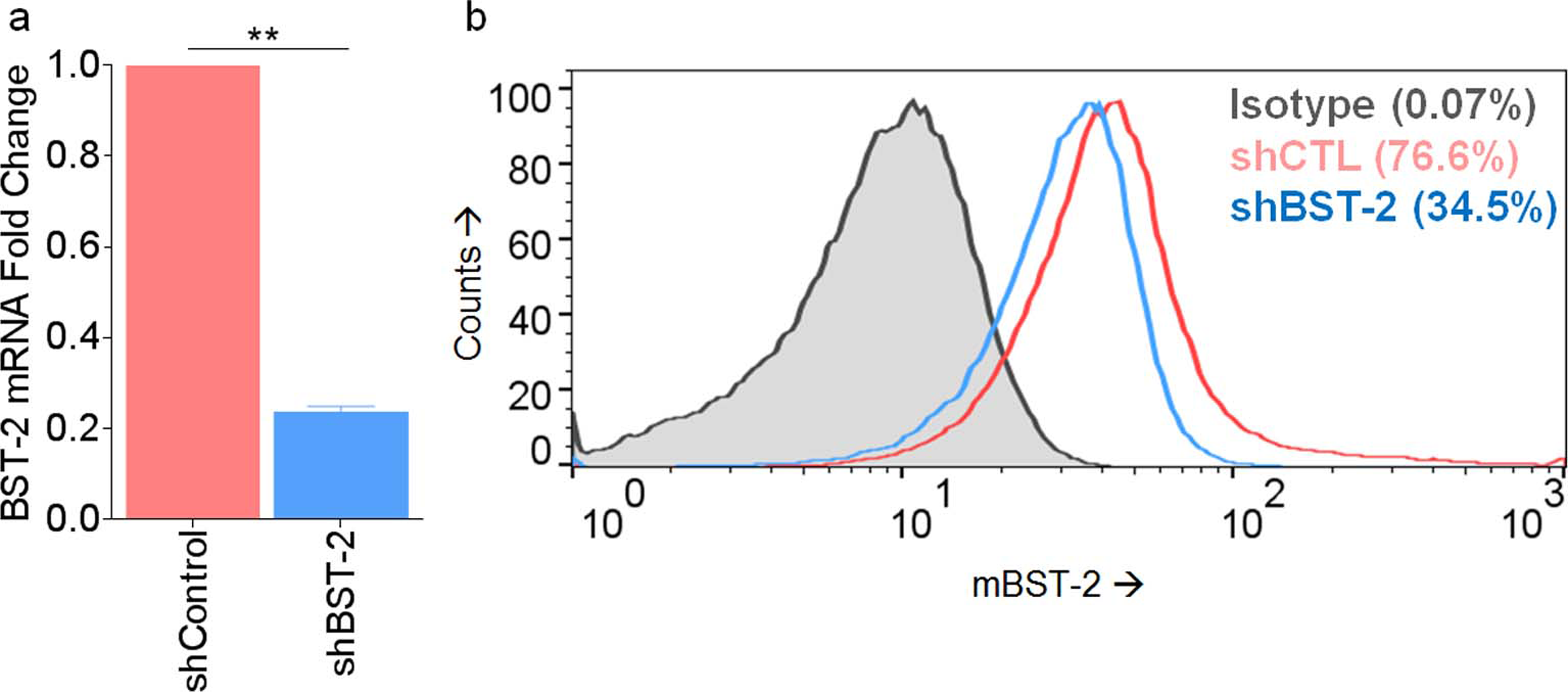

Supplement: Supplementary Figure S1 [file cddis201768x2.tif]

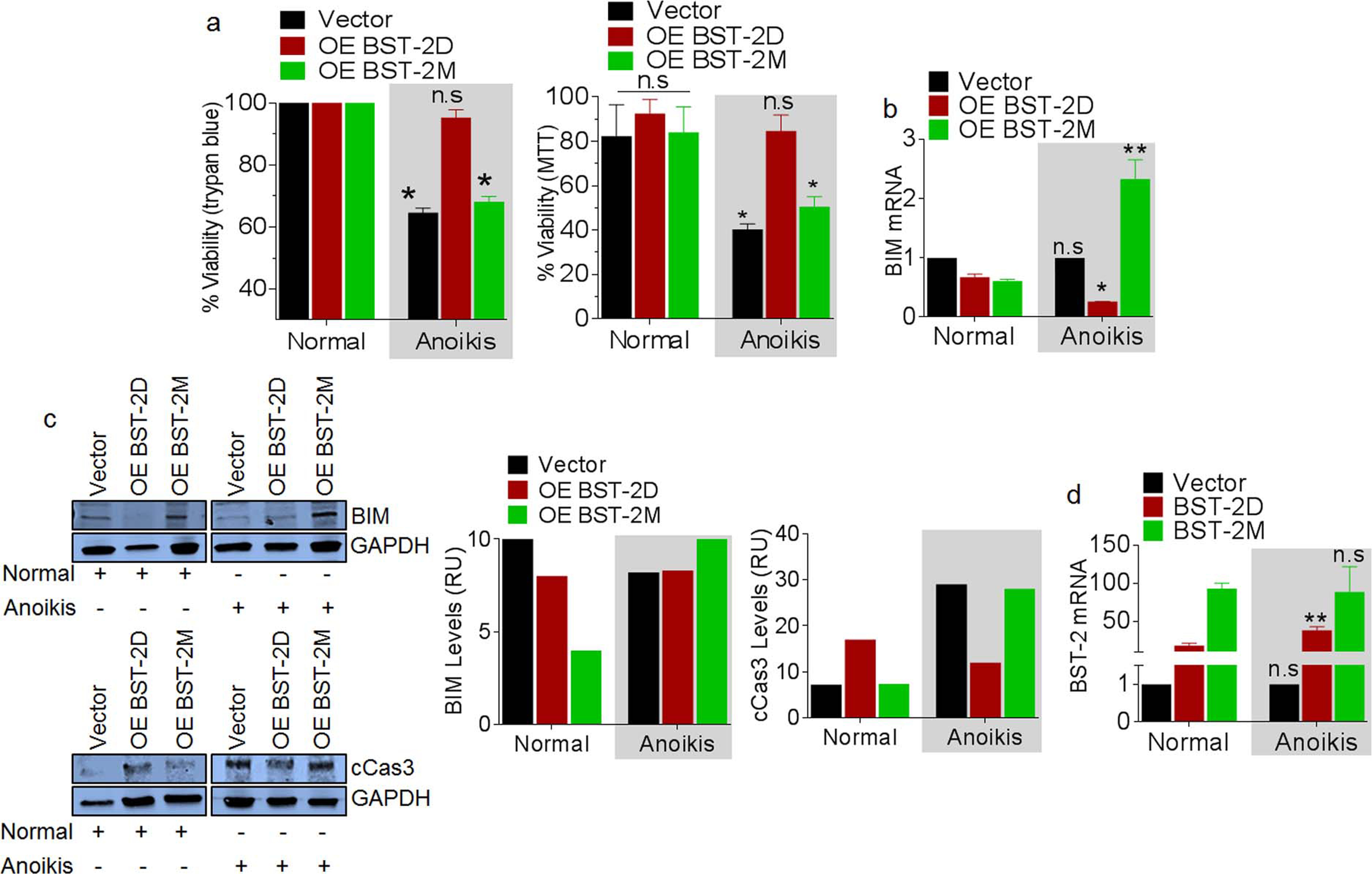

Supplement: Supplementary Figure S2 [file cddis201768x3.tif]
